# Flower-visiting assemblages of arable lands and orchards in the eastern Pannonian Lowland, studied with volatile traps

**DOI:** 10.3897/BDJ.14.e174461

**Published:** 2026-02-04

**Authors:** Aletta Ősz, Kálmán Szanyi, Dóra Arnóczkyné Jakab, Szabolcs Szanyi, Sándor Koczor, Miklós Tóth, István Szarukán, Antal Nagy

**Affiliations:** 1 Plant Protection Institute, Centre for Agricultural Research, Hungarian Research Network, Herman Otto u. 15., H-1022, Budapest, Hungary Plant Protection Institute, Centre for Agricultural Research, Hungarian Research Network, Herman Otto u. 15., H-1022 Budapest Hungary; 2 Faculty of Agriculture and Food Sciences and Environmental Management, Institute of Plant Protection, University of Debrecen, H-4032, Debrecen, Hungary Faculty of Agriculture and Food Sciences and Environmental Management, Institute of Plant Protection, University of Debrecen, H-4032 Debrecen Hungary

**Keywords:** phenylacetaldehyde, pollinator assemblages, alfalfa field, apple orchard, agroecosystem, pest monitoring, floral scent

## Abstract

Pollinator assemblages of different habitats are widely studied, but sampling methodologies and covering of lesser-known pollinators and other flower-visitor taxa (e.g., floriphagous species) need improvement to obtain reliable data on the structure and functioning of these communities. We assessed flower-visiting insect assemblages in arable lands and apple orchards of the eastern Pannonian Lowland using phenylacetaldehyde-based volatile traps originally developed for pest monitoring. In seven sites, 27,539 individuals belonging to 123 species and seven insect orders were recorded. On average, over 90% of the captured insects were potential pollinators, with a higher proportion in orchards (98%) than in arable lands (85%). In orchards, the dominant groups were Hymenoptera, Diptera, Lepidoptera and Neuroptera, while in arable lands, Lepidoptera were most abundant. Floriphagous and pest species were more numerous in arable lands. These results demonstrate that phenylacetaldehyde-baited traps are easy to use, standardized, and sufficiently efficient for assessing of flower-visiting assemblages. Beyond their application in pest monitoring, they can provide reliable data on the flower-visiting and pollinator assemblages in different natural and agricultural habitats.

## Introduction

Insect pollination is an essential ecological service, that plays a key role in global food production and the maintenance of biodiversity (Kevan et al. 1990, Klein et al. 2007, Aizen et al. 2009). Consequently, numerous studies have investigated pollinators in both natural and agricultural habitats (Brunet and Stewart 2010, Ambaw and Workiye 2020). However, most of these surveys primarily focus on major pollinator groups – particularly bees – while other flower-visiting insect taxa remained comparatively understudied (Cecen et al. 2008). Nevertheless, a wide range of less conspicuous or less well-known flower-visitor insect groups (e.g., lepidopterans, flies, beetles, etc.) also contribute considerably to plant pollination (Steffan-Dewenter et al. 2005, Garibaldi et al. 2013, Rauf et al. 2021, Rácz et al. 2023). In addition, taxa belong to other flower-visiting functional groups such as species feeding on nectar, pollen and/or different parts of the flowers and buds, and even their predators and parasites are also less known, and their interactions are also not revealed. Therefore, comprehensive surveys that encompass the wide spectrum of flower-visiting assemblages are essential to advance our understanding of insects-plant interactions (Biesmeijer et al. 2006, O'Connor et al. 2019).

Moreover, the methodological heterogeneity among studies often limits the comparability of results across different habitats (Thompson et al. 2021). Some sampling methods focus exclusively on particular flower-visiting taxa – such as pan traps or insect nets (Eeraerts and Meeus 2025) – while others rely on labour-intensive and frequently subjective sampling approaches, including visual observations, transect walks, or focal plant surveys (Sarma et al. 2025). These observer-based techniques require substantial field effort, depend heavily on the observer's expertise, and can underrepresent small, fast-moving, or nocturnal species (Roy et al. 2016). Their effectiveness is also stronly influenced by environmental conditions, leading to inconsistent detectability and limited comparatibility across studies (Sarma et al. 2025). This highlights the need for standardized methods that can be applied broadly in diverse ecological contexts (Kearns C. A. and Inouye D. W. 1993, O'Connor et al. 2019).

Volatile-baited traps, originally developed for monitoring agricultural pests, have undergone considerable development and methodological advancement in the last decades (Eby et al. 2013, Landolt et al. 2013, Szanyi et al. 2024). Due to their efficiency in the case of a broad spectrum of insect taxa, these traps are increasingly used in faunistic and ecological studies as standardized sampling tools (Henning et al. 1992, Andrews et al. 2007, Nagy et al. 2023a, Nagy et al. 2023b, Szanyi et al. 2024). Their advantages include continuous sampling independent of observer bias, high efficiency in capturing both dominant and less conspicuous taxa, and applicability across various habitats.

The efficiency of volatile baited traps for attraction of lepidopteran and hemipteran pests was previously studied by our research group. During these experiments, the phenylacetaldehyde-based lures – imitating odors of flowers – proved effective not only for their target pest taxa (Szanyi et al. 2019, Szalárdi et al. 2021), but also for several non-target flower-visiting groups, including non-pest Lepidoptera (Szanyi et al. 2019, Szanyi et al. 2024), Diptera (Nagy et al. 2023b), Orthoptera (Nagy et al. 2023a), Hymenoptera (Segers et al. 2023) and Neuroptera (Szanyi et al. 2025).

In the present study, we provide data on the flower-visiting insect assemblages of agricultural habitats of the northeastern Pannonian Lowland based on the non-target catches of phenylacetaldehyde-baited traps. Additionally, we compare the composition and diversity of these assemblages living in two different agricultural habitat types – arable lands and orchards – during different flowering periods.

## Material and methods

**Study sites and sampling**

**Figure 1.**
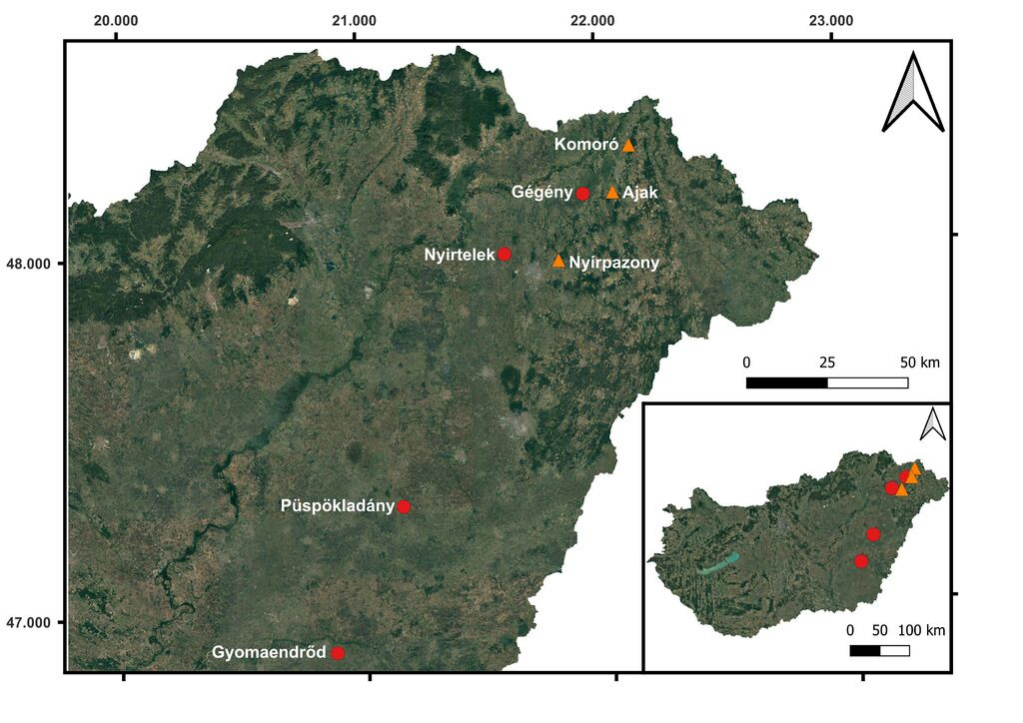
Locations of the sampling sites. Red circles=arable lands, yellow triangles=orchards.

We studied the efficiency of different phenylacetaldehyde-based lures between 2019 and 2022 at seven sites including four alfalfa fields and three commercial apple orchards in the Pannonian Lowland of Hungary (Fig. 1).

Experiments in alfalfa fields:

**Püspökladány**, Hajdú-Bihar County (GPS: 47.1814N, 21.0913E). Baits: phenylacetaldehyde, 1-phenylacetaldehyde, 4-methoxycinnamaldehyde, phenylacetaldehyde + 4-methoxycinnamaldehyde, phenylacetaldehyde + 1-phenylacetaldehyde + 4-methoxycinnamaldehyde + 1-nonanol, phenylacetaldehyde + 1,4-dimethoxybenzene, phenylacetaldehyde + benzyl alcohol + 6-methyl-5-hepten-2-one, phenylacetaldehyde + 1,4-dimethoxybenzene + benzyl alcohol + 6-methyl-5-hepten-2-one, and unbaited traps. Number of repetitions: 10. Date: from 03/07 to 31/08, 2019.

**Gégény**, Szabolcs-Szatmár Bereg County (GPS: 48.1608218N, 21.9350966E). Baits: phenylacetaldehyde, phenylacetaldehyde + anetol, phenylacetaldehyde + anisacet, phenylacetaldehyde + betion, phenylacetaldehyde + acetacid, unbaited control traps. Number of repetitions: 6. Date: from 09/07 to 31/08, 2020.

**Nyírtelek**, Szabolcs-Szatmár Bereg County (GPS: 48.000133N, 21.600674E). Baits: phenylacetaldehyde, phenylacetaldehyde + 4-methoxycinnamaldehyde, phenylacetaldehyde + 1,4-dimethoxybenzene, phenylacetaldehyde + 1,4-dimethoxybenzene, phenylacetaldehyde + 4-methoxycinnamaldehyde + 1,4-dimethoxybenzene, unbaited control traps. Number of repetitions: 7. Date: from 22/06 to 31/08, 2021.

**Gyomaendrőd**, Békés County (GPS: 46.54569N, 20.52989E). Baits: phenylacetaldehyde, phenylacetaldehyde + hexanal, phenylacetaldehyde + (E)-2-hexanal, phenylacetaldehyde + alpha-pyrene, unbaited control traps. Number of repetitions: 5. Date: from 14/07 to 31/08, 2022

Experiments in apple orchards:

**Komoró**, Szabolcs-Szatmár Bereg County (GPS: 48.292307N, 22.133233E),

**Ajak**, Szabolcs-Szatmár Bereg County (GPS: 48.163577N, 22.059229E), and

**Nyírpazony**, Szabolcs-Szatmár Bereg County (GPS: 47.978482N, 21.824846E). Investigated habitat: apple orchard. Baits: phenylacetaldehyde + eugenol + benzyl acetate, unbaited control traps. Number of repetitions: 3. Date: from 23/04 to 06/05, 2022.

**Figure 2.**
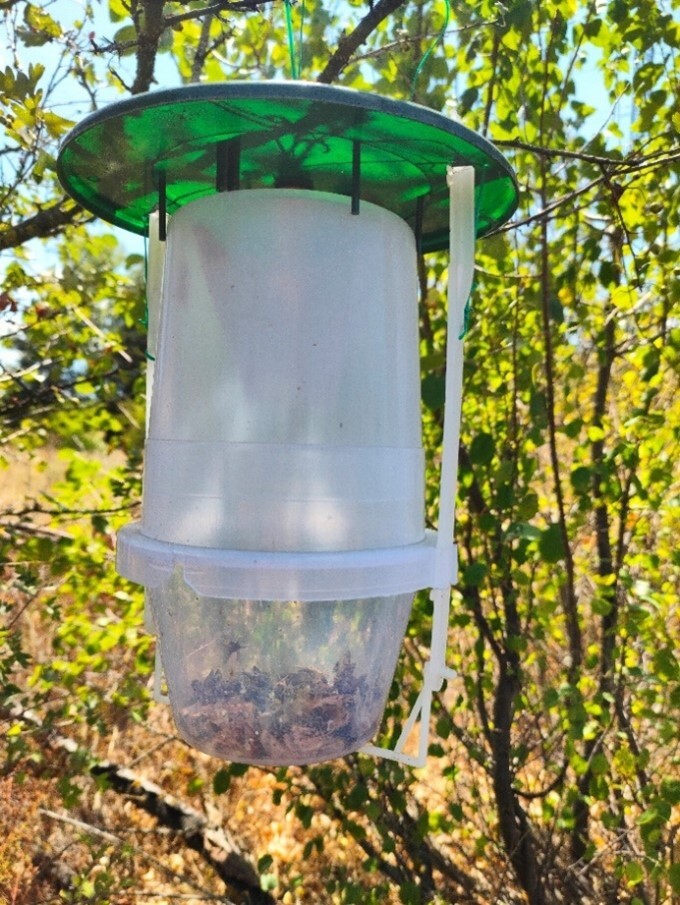
CSALOMON® VARL+ baited trap used to collect the flower visiting insects.

The samplings were carried out in the flowering period of the chosen cultures. CSALOMON® VARL+ baited traps (Plant Protection Institute, CAR, Budapest, Hungary), available at www.csalomontraps.com (accessed on 11 May 2025), were used in each sampling sites (Fig. 2.), with phenylacetaldehyde-based synthetic lures that imitate the scent of strongly fragnant, mostly white flowers (Szanyi et al. 2019, Nagy et al. 2022). The tested lures were enclosed in 1.5 × 1.5 cm polyethylene bags with a 0.2 mm wall thickness, allowing the volatiles to diffuse through the bag's walls (Tóth M. et al. 2020). In apple orchards, the traps were placed on branches of trees, with a spacing of 20 m between them at heights of 1.8–2.0 m. In alfalfa fields, traps were placed at the field margins on 1 m height mounted on wooden stakes and spaced 15 m apart. Traps were checked and emptied weekly in the apple orchards, and twice a week in the alfalfa fields. The baits were replaced every four weeks. To kill the trapped insects, we used Vaportape® II pesticide strips designed specifically for insect traps.

**Evaluation of samples and data analysis**

Samples were stored at -20 °C in a fridge until identification in a laboratory. The identification of the insects caught was made at species level, when it was possible, based on the keys of Wagner (1952) and Schwartz M. D and Foottit R. G (1998) for heteropterans, Harz (1957), Harz (1960) and Harz (1975) for orthopterans, Aspoeck et al. (1980) and Thierry et al. (1998) for neuropterans, Móczár (1985), Goulet and Hubert (1993) and Bossert (2015) for hymenopterans, Papp and Darvas (2000) and Tóth (2017) for dipterans, and Varga Z. (2010) for lepidopterans.

Relative frequencies (RF%) of insect groups were calculated using all collected specimens, including those identified only at the genus or family level. To evaluate the composition and mean ratios (MR%) of different taxonomical and functional groups, we compared both the total number and mean number of individuals (individuals/sampling sites). Two functional group classifications were applied. The first classification was based on the presumed reason attraction to floral scent: potential pollinators were considered species attracted by floral rewards for the pollination (e.g., nectar, pollen), while floriphagous species (e.g., Miridae, Cerambycidae) were those attracted due to their consumption of floral tissues. The second classification distinguished species by pest status: species capable of causing serious damage to the host plant at any phenological stage were categorized as pests, whereas those not causing such damage were considered non-pests. Data normality and variance homogeneity were assessed using Q-Q plots and Levene’s test, respectively. As assumptions were not met, the non-parametric Kruskal–Wallis test was used. Where significant differences were detected, pairwise comparisons were conducted using the Mann–Whitney U-test. All statistical analyses were performed using SPSS 21.0 (Ketskeméty et al. 2011).

All records collected for the present work were published through GBIF (Ősz et al. 2025).

## Results

A total of 30,595 insects were caught using volatile traps baited with phenylacetaldehyde-based lures (Table 1, Suppl. material 1). Of these, 27,539 individuals of 123 flower-visiting species were identified to species level, belonging to eight insect orders. Lepidoptera was notably the most species-rich order, with 98 species, and was dominated by the family Noctuidae, with 52 species of 9 subfamilies. The further most diverse groups were Hymenoptera (S=9) and Hemiptera (S=8), while Diptera, Orthoptera, Coleoptera, and Mecoptera were represented by only a few species.

Most identified species were common and widespread in Hungary. The major part of the assemblage consisted of agricultural pest species (e.g., *Hemiptera* spp., *Ostrinia nubilalis*, *Autographa gamma*, *Agrotis* spp., *Helicoverpa armigera*), primarily associated with alfalfa fields. In contrast, the majority of individuals and species caught in apple orchards were considered beneficial. Additionally, two protected species (*Bombus argillaceus*, *B. ruderatus*) were recorded in the apple orchards, and one (*Nymphalis c-album*) in the alfalfa fields.

Although Lepidoptera had the highest species-richness, they accounted for 47.25% of all specimens, with 21.75% belonging to the Noctuidae family. Within Noctuidae, the Plusiinae (RF%=53.79), and Heliothinae (RF%=37.50) subfamilies were dominant. Lepidoptera was followed by Hymenoptera (RF%=15.76), Hemiptera (RF%=6.95), and Diptera (RF%=3.64).

Notable differences were found between the flower visiting assemblages of the two habitat types. Arable lands supported a more diverse assemblage, with 24,834 specimens representing 108 species, compared to only 2,705 individuals and 22 species in the orchards.

In arable lands, Lepidoptera dominated species-richness (S=95), with other groups contributing only a few species each. However, Lepidoptera accounted for only 67.83% of individuals. Hymenoptera (RF%=15.97), Hemiptera (RF%=10.10) and Neuroptera (RF%=4.93) were also relatively abundant given their species numbers.

In orchards, only 22 species were recorded. Almost half belonged to Hymenoptera (S=9), while Lepidoptera, Diptera, and Neuroptera were represented by only a few species. Relative frequencies followed a similar pattern: Hymenoptera were dominant (RF%=53.13), followed by Diptera (RF%=34.44), Neuroptera (RF%=6.12), and Lepidoptera (RF%=6.29).

**Figure 3.**
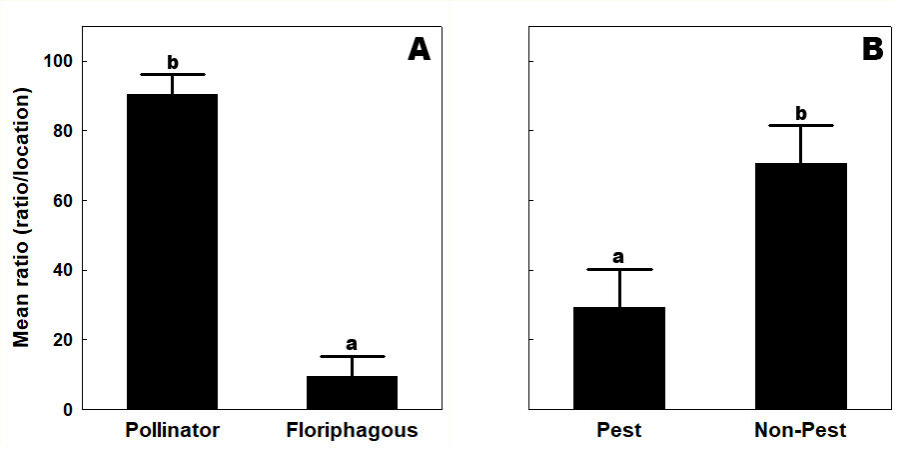
Mean ratio (ratio/location [%]) of potential pollinator, floriphagous (A), pest, and non-pest species (B). Means with the same letter within a diagram are not significantly different at P=0.05 by Mann-Whitney U non-parametric test.

Across all habitats, the mean ratio (MR%) of potential pollinators (MR%=90.462±15.049) was significantly higher than that of floriphagous insects (MR%=9.537±15.049) (Fig. 3). Similarly, the mean ratio of pest species (MR%=29.361±28.860) was significantly lower than that of non-pest species (MR%=70.638±28.860).

**Figure 4.**
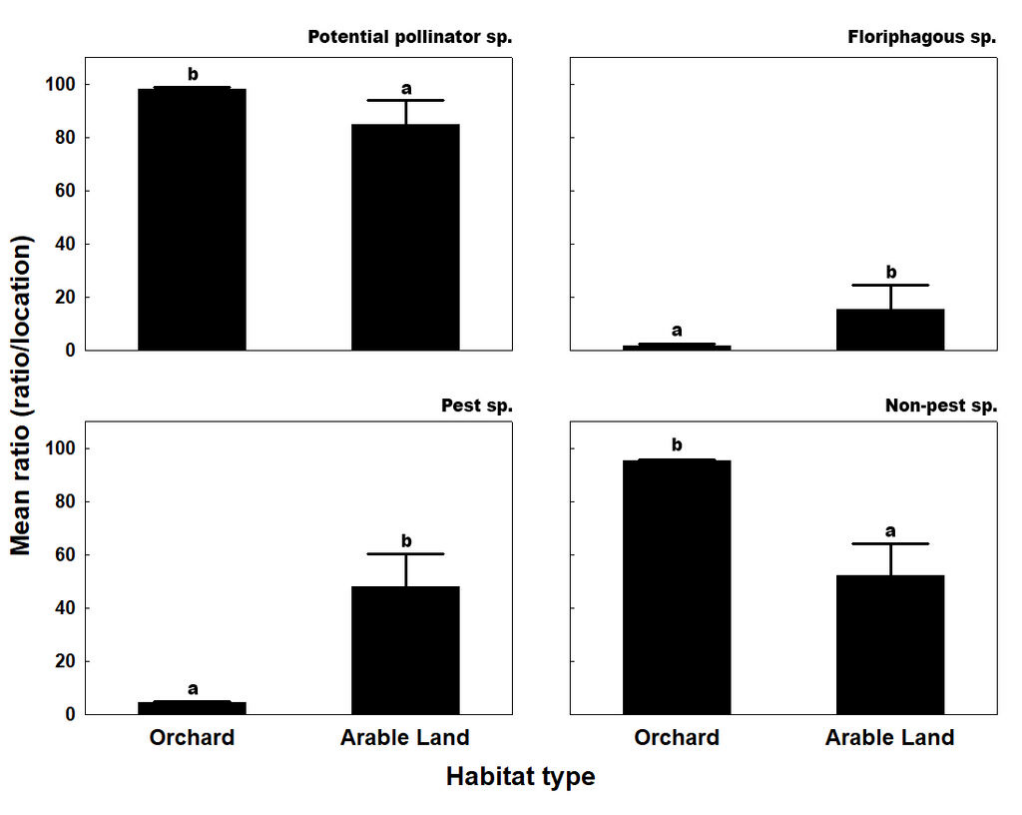
Mean ratio of potential pollinator, floriphagous, pest, and non-pest species in the different habitat types (orchard and arable land). Means with the same letter within a diagram are not significantly different at P=0.05 by Mann-Whitney U non-parametric test.

Comparing habitat types, both the mean ratio of potential pollinators (MR%=98.239±1.049) and non-pest species (MR%=95.420±0.691) were significantly higher in orchards than in arable lands (potential pollinators: MR%=84.629±18.610; non-pests: MR%=52.052±24.305) (Fig. 4). Accordingly, arable lands had a much higher ratio of pests (MR%=47.947±24.305) compared to orchards (MR%=4.579±0.691).

The dominant pollinator groups in orchards were Hymenoptera (MR%=51.537±4.275), Diptera (MR%=34.453±2.913), and Lepidoptera (MR%=6.313±0.564) and Neuroptera (MR%=5.934±2.913). In arable lands, the most abundant pollinators were Lepidoptera (MR%=63.445±28.774), Hymenoptera (MR%=15.876±27.294), and Neuroptera (MR%=4.209±6.031).

Floriphagous and pest species had significantly higher mean ratios in arable lands (floriphagous: MR%=15.371±18.610; pests: MR%=47.947±24.305) than in orchards (floriphagous: MR%=1.760±1.049; pests: MR%=4.579±0.691). The most abundant pest groups in arable lands were Lepidoptera (MR%=32.576±16.283) and Hemiptera (MR%=15.222±18.697), with the latter also comprising the only notable floriphagous group. In orchards, the most abundant pest groups were Lepidoptera (MR%=2.819±0.424) and Hymenoptera (MR%=1.760±1.049), the latter also being the sole floriphagous group.

## Discussion

A total of 123 flower-visiting insect species were recorded in arable lands and apple orchards of the eastern Pannonian Lowland. The insect assemblages included a wide range of taxa from multiple orders, reflecting the diverse communities occurring in these agroecosystems. Notably, three protected species – *Bombus argillaceus*, *B. ruderatus*, and *Nymphalis c-album* – were recorded, underscoring the ecological importance of these habitats.

The observed species richness, especially within Lepidoptera, highlights the attractiveness of phenylacetaldehyde as a floral scent. The dominance of the Noctuidae family within Lepidoptera is consistent with their known responsiveness to floral volatiles (Szanyi et al. 2019, Szanyi et al. 2024). Nonetheless, Hymenoptera and Diptera also contributed substantially, especially in orchards, suggesting that these groups play there the major role in pollination.

Our findings demonstrate that phenylacetaldehyde-based volatile traps, originally designed for pest monitoring, are highly effective at capturing a broad spectrum of flower-visiting insects belonging several orders. This supports earlier findings (Landolt et al. 2013, Hesler 2016, Nagy et al. 2023a, Nagy et al. 2023b, Szanyi et al. 2024) and highlights the potential of such traps for broader ecological and faunistic applications beyond pest surveillance.

The study revealed notable differences in the composition of flower-visiting insect communities in arable lands and orchards. Arable lands supported a more diverse assemblage, which may be partly due to the higher sampling effort and longer sampling duration in these habitats, as the flowering stage of the investigated arable crop – alfalfa – is much longer than that of the apple orchard. However, the relative frequency and ratio patterns suggest that these differences are not solely methodological. From a functional perspective, potential pollinator species made up the majority of the assemblages in both habitat types, although their proportion was significantly higher in orchards. Accordingly, pest and floriphagous species were more abundant in arable lands.

These observed differences underline the importance of multi-taxa monitoring in agroecosystems. Focusing solely on bees or model pollinators would overlook important contributors to pollination and ecosystem functioning. Moreover, the detection of protected species like *Bombus argillaceus*, *B. ruderatus* and *Nymphalis c-album* reinforces the conservation value of these habitats and the relevance of including non-target data collected with volatile traps in faunistic studies.

In conclusion, standardized volatile-baited traps can be a valuable tool for studying flower-visiting insects and their interactions with plants in various ecosystems. Their ability to provide consistent and replicable data makes them especially suitable for long-term monitoring and comparative studies in both natural and agricultural habitats.

## Supplementary Material

E6AF2E24-D2D1-545A-ACC7-C21FA441DB8A 10.3897/BDJ.14.e174461.suppl1 Supplementary material 1 Relative frequences Data type Occurences Brief description Relative frequences (RF%) of insect orders, families (only in the case of Hymenoptera and Lepidoptera orders) and subfamilies (only in the case of Noctuidae family) calculated based on all caught individuals and individual numbers of insects identified to species level. File: oo_1494994.xlsx https://binary.pensoft.net/file/1494994 Ősz A., Szanyi K., Arnóczkyné Jakab D., Szanyi Sz., Koczor S., Tóth M., Szarukán I., Nagy A.

## Figures and Tables

**Table 1.** Checklist by habitat types (A=arable land, O=orchard), including information on the occurence (1=presence), pest status (PE=pest, causes damage to crops at any developmental stage; NPE=non-pest, does not cause serious damage) and pollinator role of the species (PO=known pollinator; PPO=potential pollinator; FLO=floriphagous, feeds on the flowers). Protected species in Hungary are marked with an asterisk (*).

**Order**	**Family**	**Species**	**A**	**O**	**PE**	**PO**
Coleoptera	Cerambycidae	*Plagionotus floralis* (Pallas, 1773)	1		PE	FLO
Diptera	Bombyliidae	*Bombylius major* (Linnaeus, 1758)		1	NPE	PO
Diptera	Syrphidae	*Episyrphus balteatus* (De Geer, 1776)		1	NPE	PO
Diptera	Syrphidae	*Eristalis tenax* (Linnaeus, 1758)		1	NPE	PO
Diptera	Syrphidae	*Melanostoma scalare* (Fabricius, 1794)		1	NPE	PO
Hemiptera	Miridae	*Adelphocoris lineolatus* (Goeze, 1778)	1		PE	FLO
Hemiptera	Miridae	*Adelphocoris quadripunctatus* (Fabricius, 1794)	1		PE	FLO
Hemiptera	Miridae	*Adelphocoris reichelii* (Fieber, 1836)	1		PE	FLO
Hemiptera	Miridae	*Adelphocoris seticornis* (Fabricius, 1775)	1		PE	FLO
Hemiptera	Miridae	*Lygus gemellatus* (Herrich-Schaeffer, 1835)	1		PE	FLO
Hemiptera	Miridae	*Lygus pratensis* (Linnaeus, 1758)	1		PE	FLO
Hemiptera	Miridae	*Lygus rugulipennis* (Poppius, 1911)	1		PE	FLO
Hemiptera	Miridae	*Polymerus vulneratus* (Panzer, 1805)	1		PE	FLO
Hymenoptera	Apidae	*Apis mellifera* (Linnaeus, 1758)	1	1	NPE	PO
Hymenoptera	Apidae	*Bombus argillaceus* (Scopoli, 1763)*		1	NPE	PO
Hymenoptera	Apidae	*Bombus hortorum* (Linnaeus, 1761)		1	NPE	PO
Hymenoptera	Apidae	*Bombus lapidarius* (Linnaeus, 1758)		1	NPE	PO
Hymenoptera	Apidae	*Bombus pascuorum* (Scopoli, 1763)		1	NPE	PO
Hymenoptera	Apidae	*Bombus ruderatus* (Fabricius, 1775)*		1	NPE	PO
Hymenoptera	Apidae	*Bombus sylvarum* (Linnaeus, 1761)		1	NPE	PO
Hymenoptera	Apidae	*Bombus terrestris* (Linnaeus, 1758)		1	NPE	PO
Hymenoptera	Vespidea	*Vespa crabro* (Linnaeus, 1758)		1	PE	FLO
Lepidoptera	Crambidae	*Cydalima perspectalis* (Walker, 1859)	1		PE	PPO
Lepidoptera	Crambidae	*Haritala ruralis* (Scopoli, 1763)	1		NPE	PPO
Lepidoptera	Crambidae	*Loxostege sticticalis* (Linnaeus, 1761)	1		PE	PPO
Lepidoptera	Crambidae	*Nomophila noctuella* (Denis & Schiffermüller, 1775)	1		NPE	PPO
Lepidoptera	Crambidae	*Nymphula nitidulata* (Hufnagel, 1767)	1		NPE	PPO
Lepidoptera	Crambidae	*Sitochroa verticalis* (Linnaeus, 1758)	1		NPE	PPO
Lepidoptera	Erebidae	*Amata phegea* (Linnaeus, 1758)	1		NPE	PO
Lepidoptera	Erebidae	*Dysauxes ancilla* (Linnaeus, 1767)	1		NPE	PPO
Lepidoptera	Erebidae	*Dysgonia algira* (Linné, 1767)	1		NPE	PPO
Lepidoptera	Erebidae	*Eilema lurideola* (Zincken, 1817)	1		NPE	PPO
Lepidoptera	Noctuidae	*Euclidia glyphica* (Linnaeus, 1758)	1		NPE	PO
Lepidoptera	Erebidae	*Herminia tarsipennalis* (Treitschke, 1835)	1		NPE	PPO
Lepidoptera	Erebidae	*Hypena proboscidalis* (Linnaeus, 1758)	1		NPE	PPO
Lepidoptera	Erebidae	*Hypena rostralis* (Linnaeus, 1758)	1		NPE	PPO
Lepidoptera	Erebidae	*Lygephila craccae* (Denis & Schiffermüller, 1775)	1		NPE	PPO
Lepidoptera	Erebidae	*Lymantria dispar* (Linnaeus, 1758)	1		PE	PPO
Lepidoptera	Erebidae	*Prodotis stolida* (Fabricius, 1775)	1		NPE	PPO
Lepidoptera	Erebidae	*Rivula sericealis* (Scopoli, 1763)	1		NPE	PPO
Lepidoptera	Erebidae	*Scoliopteryx libatrix* (Linnaeus, 1758)		1	NPE	PPO
Lepidoptera	Gelechiidae	*Sitotroga cerealella* (Olivier, 1789)	1		PE	PPO
Lepidoptera	Gelechiidae	*Stomopteryx radicalis* (Falkovitsh & Bidzilya, 2003)	1		NPE	PPO
Lepidoptera	Gelechiidae	*Camptogramma bilineata* (Linnaeus, 1758)	1		NPE	PPO
Lepidoptera	Geometridae	*Chiasmia clathrata* (Linnaeus, 1758)	1		PE	PO
Lepidoptera	Geometridae	*Cidaria fulvata* (Forster, 1771)	1		NPE	PPO
Lepidoptera	Geometridae	*Ematurga atomaria* (Linnaeus 1758)	1		PE	PPO
Lepidoptera	Geometridae	*Epirrhoe alternata* (Müller, 1764)	1		NPE	PPO
Lepidoptera	Geometridae	*Idaea aversata* (Linnaeus, 1758)	1		NPE	PPO
Lepidoptera	Geometridae	*Idaea rusticata* (Denis & Schiffermüller, 1775)	1		NPE	PPO
Lepidoptera	Geometridae	*Ligdia adustata* (Denis & Schiffermüler, 1775)	1		NPE	PPO
Lepidoptera	Geometridae	*Peribatodes rhomboidaria* (Denis & Schiffermüller, 1775)	1		PE	PPO
Lepidoptera	Geometridae	*Peribatodes secundaria* (Denis & Schiffermüller, 1775)	1		NPE	PPO
Lepidoptera	Geometridae	*Plemyria rubiginata* (Denis & Schiffermüller, 1775)	1		NPE	PPO
Lepidoptera	Geometridae	*Pseudopanthera macularia* (Linnaeus, 1758)	1		NPE	PPO
Lepidoptera	Geometridae	*Tephrina arenacearia* (Denis & Schiffermüller, 1775)	1		PE	PPO
Lepidoptera	Lasiocampidae	*Lasiocampa trifolii* (Denis & Schiffermüller, 1775)	1		NPE	PPO
Lepidoptera	Lycaenidae	*Polyommatus icarus* (Rottemburg, 1775)	1		NPE	PO
Lepidoptera	Noctuidae	*Acontia lucida* (Hufnagel, 1766)	1		NPE	PPO
Lepidoptera	Noctuidae	*Acontia trabealis* (Scopoli, 1763)	1	1	NPE	PPO
Lepidoptera	Noctuidae	*Aedia leucomelas* (Linnaeus, 1758)	1		NPE	PPO
Lepidoptera	Noctuidae	*Abrostola tripartita* (Hufnagel, 1766)	1	1	NPE	PO
Lepidoptera	Noctuidae	*Abrostola triplasia* (Linnaeus, 1758)	1		NPE	PO
Lepidoptera	Noctuidae	*Agrotis bigramma* (Esper, 1790)	1		PE	PPO
Lepidoptera	Noctuidae	*Agrotis exclamationis* (Linnaeus, 1758)	1		PE	PPO
Lepidoptera	Noctuidae	*Agrotis segetum* (Denis & Schiffermüller, 1775)	1		PE	PPO
Lepidoptera	Noctuidae	*Apamea sordens* (Hufnagel, 1766)	1		NPE	PPO
Lepidoptera	Noctuidae	*Apterogenum ypsillon* (Denis & Schiffermüller, 1775)	1		NPE	PPO
Lepidoptera	Noctuidae	*Autographa gamma* (Linnaeus, 1758)	1	1	PE	PO
Lepidoptera	Noctuidae	*Axylia putris* (Linnaeus, 1761)	1		NPE	PPO
Lepidoptera	Noctuidae	*Calophasia lunula* (Hufnagel, 1766)	1		NPE	PPO
Lepidoptera	Noctuidae	*Caradrina morpheus* (Hufnagel, 1766)	1		NPE	PPO
Lepidoptera	Noctuidae	*Chrysodeixis chalcites* (Esper, 1789)	1		PE	PO
Lepidoptera	Noctuidae	*Cirrhia icteritia* (Hufnagel, 1766)	1		NPE	PPO
Lepidoptera	Noctuidae	*Cornutiplusia circumflexa* (Linnaeus, 1767)	1		NPE	PO
Lepidoptera	Noctuidae	*Cucullia chamomillae* (Denis & Schiffermüller, 1775)		1	NPE	PPO
Lepidoptera	Noctuidae	*Cucullia umbratica* (Linnaeus, 1758)	1		NPE	PO
Lepidoptera	Noctuidae	*Diachrysia chrysitis* (Linnaeus, 1758)	1	1	NPE	PO
Lepidoptera	Noctuidae	*Diachrysia stenochrysis* (Warren, 1913)	1		NPE	PO
Lepidoptera	Noctuidae	*Eupsilia transversa* (Hufnagel, 1766)	1		NPE	PPO
Lepidoptera	Noctuidae	*Euxoa temera* (Hübner, 1808)	1		PE	PPO
Lepidoptera	Noctuidae	*Hadena bicruris* (Hufnagel, 1766)	1		NPE	PPO
Lepidoptera	Noctuidae	*Hadula trifolii* (Hufnagel, 1766)	1		PE	PO
Lepidoptera	Noctuidae	*Helicoverpa armigera* (Hübner, 1808)	1	1	PE	PO
Lepidoptera	Noctuidae	*Heliothis maritima* (Draudt, 1938)	1		PE	PO
Lepidoptera	Noctuidae	*Heliothis viriplaca* (Hufnagel, 1766)	1		PE	PO
Lepidoptera	Noctuidae	*Hoplodrina ambigua* (Denis & Schiffermüller, 1775)	1		NPE	PPO
Lepidoptera	Noctuidae	*Hoplodrina octogeneria* (Goeze, 1781)	1		NPE	PPO
Lepidoptera	Noctuidae	*Hydraecia micacea* (Esper, 1789)	1		NPE	PPO
Lepidoptera	Noctuidae	*Lacanobia oleracea* (Linnaeus, 1758)	1		PE	PPO
Lepidoptera	Noctuidae	*Macdunnoughia confusa* (Stephens, 1850)	1	1	PE	PPO
Lepidoptera	Noctuidae	*Mamestra brassicae* (Linnaeus, 1758)	1		PE	PPO
Lepidoptera	Noctuidae	*Mesapamea secalis* (Linnaeus, 1758)	1		PE	PPO
Lepidoptera	Noctuidae	*Mesoligia furuncula* (Denis & Schiffermüller, 1775)	1		NPE	PPO
Lepidoptera	Noctuidae	*Mythimna albipuncta* (Denis & Schiffermüller, 1775)	1		NPE	PPO
Lepidoptera	Noctuidae	*Mythimna l-album* (Linnaeus, 1767)	1		NPE	PPO
Lepidoptera	Noctuidae	*Mythimna pallens* (Linnaeus, 1758)	1		PE	PPO
Lepidoptera	Noctuidae	*Mythimna vitellina* (Hübner, 1808)	1		PE	PPO
Lepidoptera	Noctuidae	*Noctua interposita* (Hübner, 1790)	1		PE	PPO
Lepidoptera	Noctuidae	*Noctua janthina* (Borkhausen, 1792)	1		PE	PPO
Lepidoptera	Noctuidae	*Noctua pronuba* (Linnaeus, 1758)	1		PE	PPO
Lepidoptera	Noctuidae	*Orthosia cruda* (Denis & Schiffermüller, 1775)		1	PE	PPO
Lepidoptera	Noctuidae	*Pyrrhia umbra* (Hufnagel, 1766)	1		NPE	PPO
Lepidoptera	Noctuidae	*Rhyacia simulans* (Hufnagel, 1766)	1		NPE	PPO
Lepidoptera	Noctuidae	*Shargacucullia verbasci* (Linnaeus, 1758)	1		NPE	PPO
Lepidoptera	Noctuidae	*Thalpophila matura* (Hufnagel, 1766)	1		NPE	PPO
Lepidoptera	Noctuidae	*Trachea atriplicis* (Linnaeus, 1758)	1		NPE	PPO
Lepidoptera	Noctuidae	*Tyta luctuosa* (Denis & Schiffermüller, 1775)	1		NPE	PPO
Lepidoptera	Noctuidae	*Xestia c-nigrum* (Linnaeus, 1758)	1		PE	PPO
Lepidoptera	Noctuidae	*Xestia xanthographa* (Denis & Schiffermüller, 1775)	1		NPE	PPO
Lepidoptera	Nymphalidae	*Araschnia levana* (Linnaeus, 1758)	1		NPE	PO
Lepidoptera	Nymphalidae	*Coenonympha pamphilus* (Linnaeus, 1758)	1		NPE	PO
Lepidoptera	Nymphalidae	*Nymphalis c-album* (Linneus, 1758)*	1		NPE	PO
Lepidoptera	Pieridae	*Colias croceus* (Geoffroy in Fourcroy, 1785)	1		NPE	PO
Lepidoptera	Pyralidae	*Etiella zinckenella* (Treitschke, 1832)	1		PE	PPO
Lepidoptera	Pyralidae	*Hypsopygia costalis* (Fabricius, 1775)	1		PE	PPO
Lepidoptera	Pyralidae	*Ostrinia nubilalis* (Hübner, 1796)	1		PE	PPO
Lepidoptera	Pyralidae	*Salebria semirubella* (Scopoli, 1763)	1		PE	PPO
Lepidoptera	Sesiidae	*Synanthedon vespiformis* (Linnaeus, 1761)	1		NPE	PO
Mecoptera	Panorpidae	*Panorpa communis* (Linnaeus, 1758)	1		NPE	PPO
Orthoptera	Acrididae	*Calliptamus italicus* (Linnaeus, 1758)	1		PE	FLO
Orthoptera	Gryllidae	*Oecanthus pellucens* (Scopoli, 1763)	1		NPE	PPO
Orthoptera	Tettigoniidae	*Tettigonia viridissima* (Linnaeus, 1758)	1		NPE	PPO
